# The impact of social determinants of health on early childhood development: a qualitative context analysis in Iran

**DOI:** 10.1186/s12889-022-13571-5

**Published:** 2022-06-08

**Authors:** Omolbanin Atashbahar, Ali Akbari Sari, Amirhossein Takian, Alireza Olyaeemanesh, Efat Mohamadi, Sayyed Hamed Barakati

**Affiliations:** 1 Department of Public Health, Sirjan School of Medical Sciences, Sirjan, Iran; 2 Department of Health Management and Economics, School of Public Health, Tehran University of Medical Sciences, Tehran, Iran; 3 National Institute for Health Research, Tehran University of Medical Sciences, Tehran, Iran; 4 Department of Global Health and Public Policy, School of Public Health, Tehran University of Medical Sciences, Tehran, Iran; 5 Health Equity Research Centre (HERC), Tehran University of Medical Sciences, No. 70, Bozorgmehr Ava., Vesal St., Keshavars Blvd, Tehran, 1416833481 Iran; 6 Population, Family and School Health Office, Ministry of Health and Medical Education, Tehran, Iran

**Keywords:** Early childhood development, Social determinants, Context, Policy analysis, Health policy, Equity, Inequality

## Abstract

**Background:**

Social determinants have a significant impact on children’s development and their abilities and capacities, especially in early childhood. They can bring about inequity in living conditions of children and, as a result, lead to differences in various dimensions of development including the social, psychological, cognitive and emotional aspects. We aimed to identify and analyze the social determinants of Early Childhood Development (ECD) in Iran and provide policy implications to improve this social context.

**Methods:**

In a qualitative study, data were collected through semi-structured interviews with 40 experts from October 2017 to June 2018. Based on Leichter’s (1979) framework and using the deductive approach, two independent researchers conducted the data analysis. We used MAXQDA.11 software for data management.

**Results:**

We identified challenges related to ECD context in the form of 8 themes and 22 subthemes in 4 analytical categories relevant to the social determinants of ECD including: Structural factors (economic factors: 6 subthemes, political factors: 2 subthemes), Socio-cultural factors (the socio-cultural setting of society: 6 subthemes, the socio-cultural setting of family: 4 subthemes), Environmental or International factors (the role of international organizations: 1 subtheme, political sanctions: 1 subtheme), and Situational factors (genetic factors: 1 subtheme, the phenomenon of air pollution: 1 subtheme). We could identify 24 policy recommendations to improve the existing ECD context from the interviews and literature.

**Conclusion:**

With regard to the challenges related to the social determinants of ECD, such as increasing social harms, decreasing social capital, lack of public awareness, increasing socio-economic inequities, economic instability, which can lead to the abuse and neglect of children or unfair differences in their growth and development, the following policy-making options are proposed: focusing on equity from early years in policies and programs, creating integration between policies and programs from different sectors, prioritizing children in the welfare umbrella, empowering families, raising community awareness, and expanding services and support for families, specially the deprived families subject to special subsidies.

**Supplementary Information:**

The online version contains supplementary material available at 10.1186/s12889-022-13571-5.

## Background

There is a lot of evidence that shows vital development in children begins before their birth and continues in the first 8 years of life [1]. In various literatures, this period is considered the most critical period in human life because it is the fastest period of brain development [2]. Also, it is the most cost-effective period of life to invest in the development of human capital [3]. However, an important consideration in this regard is that early childhood development (ECD) is not only affected by heredity but there are also numerous variables in the child’s living environment at the micro, meso, exo and macro levels which play an important role in ECD [4].

Among the factors that can affect ECD, the following can be mentioned: education of parents [5], maternal mental health [6, 7], malnutrition, infectious diseases, exposure to environmental toxins [8], limitations of intrauterine growth [7], ethnicity [9], characteristics of family environment [10], quality of child care [7, 11], parent-child interactions [12], socio-cultural context, biological factors, and genetic inheritance [5], child’s educational opportunities or cognitive motivators, and exposure to violence [7]. On the other hand, children’s failure to realize their developmental potential plays an important role in the intergenerational transmission of poverty [5]. The fact is that more than 200 million children in developing countries are failing to reach their developmental potential [13].

Given the nature of ECD, the issue of inequity has a particular importance, since unequal conditions and opportunities in society will have adverse effects on the development of children’s capacities and abilities in various social, psychological, emotional, and physical aspects [14]. Inadequate and unequal living conditions are the result of deeper structural factors that together shape the way societies are organized with inappropriate social programs and policies, unfair economic conditions, and inappropriate policies. In this regard, the new global agenda on health equity states that Our children have dramatically different opportunities to live, depending on where they are born. In Japan or Sweden, they can expect to live more than 80 years; in Brazil 72 years, in India it is 63 years and in one of several African countries, it is less than 50 years [15]. Between and inside the countries, there are huge differences in the chance of survival, and this can be seen all over the world. In many countries, at all income levels, the development of children and the outcomes of children and families follow a social gradient: the lower the socio-economic conditions are, the poorer the children’s pertaining conditions will be, and finally, the more unfavorable developmental status they will have. In this regard, and as reflected in a report by the World Health Organization’s Commission for Social Determinants of Health, (2008), entitled “Closing the Gap in a Generation”, ECD has been emphasized [16]. Also, in the sixth chapter of the Health in All Policies report entitled “ Seizing opportunities, implementing policies “ published by the Ministry of Social Affairs and Health of Finland in 2013, the promotion of equity from the start through the ECD and health has been focused on in all policies [17].

In Iran, after the setting up of a Committee for Social Determinants of Health and the selection of ECD as one of its highest priority subjects, it was proposed that a policy document on ECD be drawn up by the Ministry of Health, the Ministry of Education and the Welfare Organization in 2008. Nevertheless, this policy document has not been implemented at a national level [18]. As experts and policy makers in the field of children have reached a consensus, the current situation of Iranian society at all levels of ECD policy making is facing many challenges [19] such as the lack of integration and coordination between policies and programs in various sectors, the lack of focus on all aspects of ECD, and lack of emphasis on eliminating the existing inequities [20]. These problems regarding children’s development in the country have prevented them from fully achieving their rights [21].

In relation to child health, according to Iran’s Multiple Indicator Demographic and Health Survey (IrMIDHS, 2010), the rate of underweight and short stature in children has been reported to be 4.8 and 6.83%. Also, a heterogeneous distribution of child malnutrition at the national level with high prevalence in deprived provinces was seen [22]. The sampling method in this survey was multi-stage stratified cluster-random. The final sample size in this study was 31,350 households in the country. Information on sampling clusters was obtained from the Statistics Center of Iran [23]. Based on the national survey of anthropometric indices in children under 5, which was conducted in 2017, the percentage of underweight and short stature was reported to be 4.3 and 4.8% respectively and a significant difference was seen between urban and rural areas. In this study, the sample size is 600 children for each province and the data obtained from health ministry software (SIB software) were selected randomly [24]. Various studies have also shown a reduction in child mortality rates in Iran in recent decades [22, 25–28]. In this regard, in the IrMIDHS survey (2010), the mortality rates of under-5 children, infants and neonates per thousand live births were reported to be 22.52, 20.32 and 15.29 respectively and there were differences among various provinces of the country [22]. Moreover, based on Hosseinpoor’s (2005 and 2006.) studies, a significant difference was seen in infant mortality rate between various provinces and the lowest and highest socioeconomic quintiles [25, 26]. In these studies, data extracted from the Iran’s Demographic and Health Survey (DHS), which was conducted in 2000 [28]. The concentration index of infant mortality was used to measure the socioeconomic inequalities [25, 26].

On the other hand, in relation to child education and according to the Educational Inequality Index (UNDP) (2014), Iran ranks 12th in the region with a score of 0.433 followed by Syria, Iraq, Pakistan and Afghanistan [29]. Based on the report from the Social Welfare Studies Office, some reasons for the increase of inequality in education include the neglect of the quality of manpower in education, non-compulsory and non-free preschool education, failure to provide statistical reports on indicators of educational inequality by the government, and no attention paid to the field of education by civil society [30]. Given that, no study has so far been conducted in Iran to examine the policy context of ECD. Also, with regards to the importance of the early years in human capital development and sustainable development of the society as well as the critical role of social determinants in ECD, the study aims to identify and clarify the contextual factors affecting ECD and its policy process in order to identify policy recommendations to improve the current situation. The context refers to the circumstances and settings in which children are born and raised. It includes systematic economic, political, social, and cultural factors at national and international levels which may influence the ECD [31]. This study answers the following questions: What factors (including structural, situational, social, economic, political, and international) affect ECD and policymaking in various levels of micro, meso, exo and macro in Iran? What works to decrease the existing inequities and improve the context for optimal ECD?

## Methods

### Conceptual framework

In this qualitative study, the researchers attempted to explore the ECD context of Iran. We used the Leichter (1979) conceptual framework for policy context analysis. This divided contextual factors into four categories including: situational factors (irregular and unstable events such as war), cultural factors (values ​​of society or different groups in society), structural factors (more stable factors of social organizations such as economic-political system) and environmental factors (factors outside the national system of politics such as multinational corporations) [32]. To collect the data, the researchers made use of interviews with experts from different sectors related to children including health and nutrition, early care and education, and protection.

### Data collection

Forty face-to-face, in-depth, semi-structured interviews were conducted from October 2017 to June 2018 using an interview guide (Appendix 1) in Tehran, Iran. Since no new data was added to our study during the last interviews, we concluded that the data has reached the saturation level. All interviews took place in the interviewees’ workplaces and each interview lasted for 30–90 minutes.

### Sampling method

To select the participants, we used the purposive sampling approach with maximum variation in terms of scientific background, activity domain, employment status, gender, and executive experience. In addition, the snowball sampling method was used to identify more interviewees. The participants were divided into five groups including policymakers (PM), managers (M), academics and researchers (Aca), NGOs’ representatives (NGO-R), and children service providers (CSP) from different organizations related to ECD (Ministry of Health and Medical Education; State Welfare Organization; Ministry of Education; Ministry of Cooperatives, Labor, and Social Welfare; Ministry of Justice; Children’s Medical Center; The Islamic Consultative Assembly; Society for Protecting the Rights of the Child (SPRC); universities and research centers, etc.) (Appendix 2). The participants met at least one of the following criteria:

- Specializing in majors related to children or neuroscience, social sciences, human sciences, and rehabilitation sciences
- Having at least 3 years of professional experience with children in non-governmental or governmental sectors
- Having a position related to children’s affairs in non-governmental or governmental sectors at the time of the study (Appendix 2)

### Data analysis

For data analysis, a deductive approach was used. In this regard, the interviews were transcribed verbatim, the codes were extracted from the summaries of the interviews, the open coding was carried out, and the extracted codes were finally categorized based on Leichter’s (1979) framework [32] using the thematic analysis approach. Coding and data categorization were done manually. MAXQDA.11 software was also used to assist data management. To ensure the accuracy of statements, transcripts were sent to the participants who were asked to confirm if necessary It should be noted that in the meantime, no changes were made to the transcripts. AA and OA also analyzed the data separately and then cross-checked the extracted themes and sub-themes, discussing the differences among some of the obtained themes and sub-themes and reaching a consensus. The consensus was then finalized by two team supervisors (AT, EM) and the confirmation was made by cheking these changes to ensure the validity of the qualitative analysis and the consistency of the findings among the authors.

### Ethical considerations

Before the interviews, necessary information regarding the study and its objectives were given to the participants and informed consent was obtained from them verbally. Moreover, they were assured that their information would remain confidential and the data of the study would be analyzed anonymously. Also, the current study has been confirmed by the Ethical Committee of Tehran University of Medical Sciences (IR.TUMS.REC.1396.2694).

## Results

The results of this study are presented based on Leichter’s (1979) framework under analytical categories including structural, socio-cultural, environmental (international), and situational factors. In our study, 8 themes and 22 subthemes were identified (Tables 1,2,3 and 4). These categories are presented in the following:

**Table 1:** Structural factors affecting ECD

Themes	Subthemes	examples	Relation
**Economic factors**	Decreased economic growth	- Increasing poverty, unemployment, etc., all of which reduce the well-being of children	(PM 9)
		- Reducing per capita budget for health, education, etc.	
		- Increasing household expenses	(M 6)
		- Reducing family investments for children	(PM 8)
		- Negative effects on the quality of parenting, the formation of behavior, and mental and physical development of children	(CSP 4)
	Economic instability	- Increasing parents’ financial stress and its negative effects on relationships with their children and reducing life quality among families and children	(Aca 3)
	Reverse country investment curve	- Low return on investment due to the highest investment in youth and middle age and the lowest investment in childhood in the country	(PM 9)
		- No allocation of a certain amount of GDP to children	(PM 38)
	Economic inequities	- Inequity in access to available resources, services and care for children in different families	(Aca 24)
		- Creating a significant difference in the chances of achieving potential development in children in different families	(PM 8)
	Inflation	- Decreased level of welfare of families and children due to rising prices of basic necessities of life such as food, clothing, and housing as well as ignoring some of the needs of children	(M 27)
	Family living conditions	- Poor economic status at the micro level, which reduces the standard of living for children, the inability to meet the natural needs of children, the increase in the number of working children, the reduction in school enrollment, and the negative effects of low quality housing on the formation of behavior and mental and physical development of children, and vice versa.	(CSP23)
**Political factors**	Lack of political discourse	- Lack of a comprehensive and coherent discourse about children in the country	(PM22)
		- Lack of space for negotiation, confusion of negotiation processes, and lack of mutual dialogue between institutions	(PM12)
	Different interpretations by different intellectual and political groups in relation to ECD	- Political decisions related to children’s affairs and influencing the approaches used for the integrated development of children	(PM25)

**Table 2:** Socio- cultural factors affecting ECD

Themes	Subthemes	examples	Relation
**The socio-cultural setting of society**	Social inequities	- Inequity in children’s access to quality health services, nutrition, care and education in early childhood and significant differences in children’s chances of achieving potential development	(NGO-P 28)
	Uneven urban development	- Consequences of a violent city, the spread of marginalization and the impacts of an insecure environment on the physical, psychological and social development of children	(PM32)
		- Change of lifestyle, increasing social harms and their negative consequences for children’s development	(Aca26)
		- Lack of play spaces and reduced child-friendly environments	(CSP18)
	Decrease in social capital	- Reducing social stability and cohesion, social cooperation and participation and its negative effects on family stability	(PM10)
		- Reducing social and family relationships and its impact on children’s development	(M17)
	Misconceptions and ignorance of society about ECD	- Lack of awareness and knowledge about the importance of ECD, it’s various dimensions and the influential factors	(PM31)
		- Existing misconceptions in society about nurturing methods such as using physical punishment	(NGO-P 11)
	Development of communication technology and media	- Increasing parents’ awareness and knowledge about the importance of childhood and proper nurturing methods	(M17)
		- Increasing the need for media literacy in parents to identify correct and incorrect information as well as managing children’s use of technology	(Aca37)
		- The positive and negative effects of using technology on children’s development	(CSP16)
	Issues in the national educational system	- Lack of attention to sustainable and comprehensive development of children in the education system	(PM 15)
		- Relying solely on the transfer of knowledge in the education system and not addressing the development of children	(M 27)
		- Insufficient attention to teaching life skills and plays in kindergartens and primary schools	(PM13)
		- Having children who have dropped out of school as well as the fact that not all children are covered in preschool programs	(PM36)
**The socio-cultural setting of family**	Social-demographic factors of family	- Affecting the quality of life of children and their access to health care services, adequate nutrition, quality care and education in early childhood by demographic variables of families such as parental education	(PM 19)
	parenting style	- Nurturing methods and theirs negative or positive effects on the formation of the child’s personality and poor or appropriate physical, psychological, emotional, social, cognitive and spiritual development of children	(NGO-P2)
	Information and educational poverty of children in the family	- Lack of awareness in children about their rights, life skills, how to solve problems, how to communicate with others, etc.	(CSP35)
	Family harms	- Development of various mental disorders in children such as fear and anxiety, feelings of insecurity, depression, aggression, guilt, pessimism, etc. due to problems in the family and the formation of a negative and insecure parent-child relationship	(CSP30)

**Table 3:** Environmental (International) factors affecting ECD

Themes	Subthemes	examples	Relation
**The role of international organizations**	International programs	- Affecting international policy frameworks that increase the commitment and accountability of countries to their actions related to children towards the international community	(PM36)
		- International financial and technical assistance for developing countries to meet their obligations	(M14)
		- Creating international competitions among countries in order to be in a good position in international evaluations with regard to indicators related to children’s affairs	(PM13)
**Political sanctions**	Violation of the rights of Iranian children in various fields of health, nutrition, security, etc.	- Facing the problem of importing required goods and materials in the different field such as medicine, medical equipment, etc., and its negative effects on the development of Iranian children	(PM20)

**Table 4:** Situational factors affecting ECD

Themes	Subthemes	examples	Relation
**The role of international organizations**	**Genetic factors**	- Increasing developmental disorders and the costs of treatment and rehabilitation for the family, the health system, and the education system	(CSP18)
**Political sanctions**	**The phenomenon of air pollution**	- Negative effects of air pollution on physical and mental development of children in metropolitan areas	(PM40)

### Structural factors

In this analytical category, two themes and eight sub-themes were identified.

#### Economic factors

The participants stated that economic factors change the well-being of children through various ways. These factors can directly affect children’s well-being and development by increasing or decreasing family financial resources. Indirectly, these factors can affect government revenues and the sustainability of government resources to provide beneficial services to children. Although the living conditions of the family are affected by the macroeconomic status of the country, even in prosperous and developed countries, there are deprived and poor families. Therefore, it is necessary to consider the economic status of families separately. In this study, factors such as parents’ employment status, family income and housing status have been identified as indicators of family living conditions. Also, the concept of economic inequities was repeatedly cited by experts in the interviews as one of the most important factors influencing children’s opportunities for optimal development and ultimately the continuous vicious cycle of poverty.

*“Well, when you compare countries, you certainly see countries that have high economic growth and their economic situation is better, the quality of life of their children is higher, and they receive the high-quality services* (CSP 4)*.”*

*“If the family is suffering from issues such as poverty and unemployment, this family will not be able to take positive actions in this regard, no matter how much you talk about the development of the children and increase the knowledge of that family* (PM10)*.”*

*“If you look at the investment curve of the country, it is a linear curve, which is the complete opposite of opportunity. We have the highest investments in the third, fourth and fifth decades of human life and the resources are spent there, while milestones of development are established in early years* (PM9)*.”*

*“ Inequities and gaps between the rich and the poor in the country and the problems caused by the poverty of families such as the phenomenon of working children, addiction, child abuse, etc. exacerbate the issue* (PM 8)*.”*

#### Political factors

Political factors were one of the important issues which have a significant impact on ECD and the design of programs and policies in this regard. In this category, participants pointed to the politicization and policy-based decisions made by these streams of thought and politics.

*“The fact is that our political and national discourse on children only goes back to school education. That means we do not have a very coherent, comprehensive discourse on children* (PM 22)*.”*

*“One of the main problems that we face in the field of children, which is perhaps less seen, is the contradictory political attitudes and thoughts that we have towards family, women and children* (PM 12)*.”*

### Socio-cultural factors

In this analytical category, two themes and ten sub-themes were identified.

#### The socio-cultural setting of society

Another point that was mentioned in the interviews as an important factor in ECD was the socio-cultural factors of society. According to the findings of this study, the socio-cultural context of society affects ECD directly and indirectly (by influencing the socio-cultural context of the family). In this study, the socio-cultural context of society has been identified by several concepts including social inequalities, uneven urban development, declining social capital, misconceptions and ignorance of society, development of communication technology and media, and issues in the national educational system. In several interviews, for instance, the uneven development of urbanization was mentioned as a social-cultural factor which has made cities an unsafe and unfriendly environment for children. Participants stated that the uneven development of urbanization is associated with many consequences, including increasing social harm, expanding marginalization, changing lifestyles, creating a harsh city, air pollution, noise pollution, etc., each of which will affect ECD in special ways. Another concept that was mentioned as an effective factor in children’s development was the social capital. In other words, the interviewees referred to the negative impacts of decreased social capital on children’s personality by decreasing public trust and weakening empathy, social responsibility, and identity.

*“How many of us are marginalized now? Statistics say that we have twelve million marginalized people, some say eighteen million, right? What does marginalized mean? That is, those who do not receive education, care and health facilities that are necessary for the growth and development of their children?* (CSP 18)*.”*

*“Social capital in our society has decreased, distrust is too much; this affects how the child's personality is formed* (M 17)*. “*

*“Many parents, people and teachers still consider physical and verbal punishment as correct methods of nurturing while these are examples of child abuse* (Aca 4)*.”*

#### The socio-cultural setting of family

The family plays an important role in the well-being and development of children. Parental behavior and family environment can promote or inhibit children’s development. In this study, factors such as socio-demographic variables of the family have been identified as effective factors in ECD in relation to the socio-cultural context of the family. For example, many participants referred to family harms including various types of domestic violence (such as physical, sexual, psychological, and verbal violence and indifference), mental problems of parents (such as stress, anxiety, depression), parental conflict, separation or loss of parents) as factors influencing children’s development. Another very important factor that was repeatedly mentioned in the interviews was the knowledge, attitude and practice of parents regarding parenting. In other words, parenting style was believed to have a tremendous impact on the formation of children’s personality and development. In several interviews, the weakness of families in nurturing their children has been mentioned. Issues which were mentioned in connection with the role of parenting included nurturing of dependent and non-capable children, children with inability to say no, those with inability to solve problems and those who lack social skills. Such issues also included nurturing children with emotional deficiencies and mental problems as well as children who will face academic failure and social harms in the future.

*“I think we have some defects in parenting. With my experience in psychology, we do not have many independent children. Or we sometimes see that they do not have the ability to say no or their problem-solving abilities are weak. There are a number of nurturing problems* (Aca 26)*. “*

*“Domestic violence, from physical and verbal violence to other types of violence in our country, is at a high rate. Research has shown that children have this experience in terms of psychology* (PM 13)*.”*

*“Our children have little information about their rights. Families should provide some information and lessons to their children, but we see that families do not even educate their children about healthy behaviors* (NGO-P 11)*.”*

### Environmental (international) factors

In this analytical category, two themes and two sub-themes were identified.

#### The role of international organizations

Among the interviews, the role of international organizations including UNESCO, UNICEF, the World Bank, and the World Health Organization was mentioned. Interviewees stated that the political commitment of international organizations to ECD could play an important role in facilitating national political commitments to young children. Interviewees cited the financial and technical support of these international organizations to the countries.

*“International programs also have an impact on the national context of countries, such as the Sustainable Development Goals, which include seventeen core programs, some of which are directly related to health and some are directly related to poverty alleviation.” “It can also have a positive effect on our country so that we can finally pay more attention to these programs* (Aca 1)*. ”*

#### Political sanctions

Another issue raised by some interviewees was political sanctions as an external and international factor influencing the conditions and well-being of society, including the development of children.

*“Well, now these sanctions that we are facing act as a lever of pressure and disturb the condition in the country, so that they can have many effects on different people in society and lead to the violation of the rights of people, including children* (PM33)*.”*

### Situational factors

In this analytical category, three themes and three sub-themes were identified.

#### Genetic factors

Genetic factors are mentioned as a situational factor in interviews with health policy makers and health service providers. They stated that development is the result of a combination of both environmental and genetic factors. Therefore, genetic factors can be the source of some developmental disorders and defects in children.

*“Children's development is affected by various factors, including psychological, social, hereditary and environmental factors, so we can say that children's development is the result of a dynamic and continuous interaction of biological and acquired factors* (CSP 18)*.”*

#### The phenomenon of air pollution

Air pollution in some regions of the country was another situational factor mentioned in our study. This phenomenon has adverse effects on the physical and mental development of children as one of the vulnerable and sensitive groups in the society. It has also been stated that children living in societies with low socioeconomic status are more likely to be exposed to toxic waste, air pollution, poor water quality, excessive noise, and poor housing quality.

*“Well, look at the problem of air pollution and dust, which can have an impact on the health of society, especially pregnant women and children. Some of these problems manifest themselves in the short term, such as shortness of breath, allergies, asthma, and some manifest themselves in the long term.* (PM 40)*.”*

*“The effects of air pollution, noise pollution, and poor environmental quality are greater on poor children because they probably have very poor access to protective equipment and facilities* (M14)*.”*

## Discussion

According to the results of this study, economic factors can make a significant difference in children’s life conditions and affect the financial space of governments and families to invest in ECD. This issue has been emphasized in many studies. According to the World Bank, OECD member countries spend about 1.6% of their gross domestic product (GDP) on family and preschool services for children aged 0 to 6, of which 0.43% is spent on kindergartens alone. By comparison, low-income countries such as Nepal, Kenya, and Tajikistan spend only 0.1% of their GDP on preschool services, compared with less than 0.002% in Nicaragua and Senegal [33]. Based on the results of PISA study (2012), the mathematical performance of 15-year-old students in countries such as Italy, Greece, Finland, Thailand, Spain, etc., as compared to their performance in 2003, shows an increase of 25 points, which is due to an increase in the enrollment rate in preschools in this period in the mentioned countries [34]. In Iran, privatization in public education and, at the same time, a significant (50%) reduction in the share of education in the public budget are the main reasons for the increase of educational inequalities [30] so that statistics from 2011 to 2012 shows that the government’s involvement in preschool education was only for licensing. According to statistics, the number of government-run preschool centers have dropped to zero in this year [35]. World Bank’s report (2003) it was stated that the positive impact of preschool on improving education and breaking the poverty cycle can be proven in the case of Iran [30].

Economic inequities were also emphasized in our study. This shows that economic growth alone is not enough, but the distribution and quality of this growth is very important. In this regard, Boyden has emphasized the nature and quality of economic growth for ECD in his study. He states that policies should be made to ensure the sustainability of investments, to focus on the most vital stage in childhood, and to bring about benefits for all children [36]. Another study by Bennett has shown that improvements in children’s access have been distributed differently among different socioeconomic groups, and different results have been achieved [6]. Abbasian and Mahmoudi, in their study, examined the situation of child poverty in Iran. The results showed that on average between 22 and 27% of children suffered from poverty during 1983–2013. This study also showed that rural children and girls have a higher poverty rate than urban children and boys [37]. Also, according to the Social Studies and Research Institute of Iran, 34.7% of street children are hungry [38]. In the area of education, the difference in preschool coverage rates between urban and rural areas has not changed in Iran during the 1980s and 1990s, and the gap between the two areas has always been 15 to 20% [29]. For example, the coverage rate for urban and rural preschools in 2006 was 56.7 and 34.7%, respectively [30].

Another issue mentioned in this study is the role of political factors in the form of various political and intellectual mainstreams which impact the design of appropriate programs and policies for children. Since the influence of attitudes, interests, expediencies and political decisions on phenomena at the level of community is quite evident, the role of the political context in ECD has been emphasized in several studies [39–41]. Vegas states that the political context influences a country’s investment in ECD and the type of policies and programs it finances [39]. Also, Moussa’s study shows that the political violence affects the children’s mental health [41].

In our study, the effect of socio-cultural factors on ECD, like economic factors, has been considered at both macro and micro levels. The essence and quality of the social environment affect the ECD and the performance of families [42]. Among these factors, social inequalities play a critical role. The increased risk of adverse health outcomes is not limited to the lowest levels of poverty and socioeconomic status, but many child health outcomes indicate that there is a social slope. For example, birth weight indicates a specific social slope that has profound effects not only on childhood and infancy but also on adulthood [43]. Vaida argues in his study that racial and ethnical inequalities play a significant role in birth outcomes in Wisconsin. A higher proportion of infants born to black/African American women than infants born to white women are low birth weight and premature, which is the leading cause of death for black/African American infants [44]. Participants also cited the consequences of uneven urban development as lifestyle changes, increased marginalization, and social harm, all of which have negative effects on children’s development, including obesity, increased violence against children, and the creation of an insecure environment for children. Based on Jalili Moayad’s results, Iranian working children experience a relatively high rate of abuse in their work environments. 77.6% of these children have experienced at least one type of abuse, of which emotional abuse is the highest at 70.4%, followed by negligence at 52% [45].

Also, the report on the kids in communities shows that neighborhoods marked with security concerns, garbage on the streets, and delinquency were associated with a number of adverse health behaviors and consequences, including overweight and childhood obesity, behavioral problems, and other negative consequences of child development [39]. Moreover, Powers et al. emphasized the role of social capital and stated that poor social cohesion, social capital, and social support are associated with increased maternal postpartum depression, child abuse, and alcohol drinking and smoking in pregnancy [46] and potentially play a role in the current health slope among children [47]. This finding is consistent with the results of our study. In addition to addressing the social and economic causes of childhood inequities, it is important to consider cultural factors as well. In this study, cultural factors that can affect the physical and psychological development of children are referred to. These factors appear in the form of misconceptions and lack of awareness in the society with regard to parenting methods such as the belief in physical punishment in child rearing or their beliefs about the unnecessity of child sexual education. Such misconceptions have also been addressed in other studies. For example, Moore states that one of the cultural misconceptions among Australians is that young children are passive in absorbing concepts and their lives are perceived to be so simple that will not be disturbed or disrupted by influential factors. He argues that these misconceptions can indirectly increase or maintain early childhood inequities by influencing public opinions in general, and the extent of governmental support and investments in reducing early childhood inequities [47]. In Iran, negative beliefs such as avoiding to feed the baby with colostrum to prevent neonatal jaundice are seen among some ethnicities, especially those living in rural areas [48] Based on Oveisi’s study, in general, the families believe that the use of physical punishment in raising children is sometimes necessary [49]. Also, according to IRMDIS study, 18.18% of parents considered the use of physical punishment appropriate for raising a child and 79.33% used verbal punishment to raise a child [22].

Based on our findings, Families play a critical role in the well-being and development of children. Parental behavior and family environment can promote or inhibit children’s development. Because families are the first environments in which children interact with others from birth, they play a very important role in preparing children with stimulation, support and kindness. These characteristics are, in turn, influenced by the resources that families have to devote to parenting (strongly influenced by income), which is the same as their parenting style. Such characteristics tend to provide a rich and responsive environment (strongly influenced by parents’ education levels) [39, 50–52]. Hesterman states that Adverse Childhood Experiences (ACEs) such as abuse, neglect, domestic violence, discrimination, etc. and toxic stress (non- tolerable stress) threaten the physical and mental health of the child, impairing their brain development and emotional regulation. Moreover, their long-term effects are evident in adulthood [53]. In this regard, Hajnasiri in a meta-analysis study with a sample size of 15,514 on 31 articles from 2000 to 2014 estimated the prevalence of domestic violence at 66% in Iran [54] which could have adverse effects on children’s development. Still, there are not enough legislations or organizations to support these victims [55].

This study revealed that international factors, including the political commitment of international organizations to ECD, could play an important role in facilitating national political commitments to young children. Strong sponsors of ECD investment such as the UNESCO, the UNICEF, the World Bank, and the World Health Organization can provide financial support and technical advice to country leaders, including the latest evidence and the best practices. In addition, international development treaties can support national and social policies that focus on the needs of children. International policies, such as the Millennium Development Goals, offer developing countries a challenge and an opportunity. Millennium Development Goals are very child-centered, with a strong focus on children and synergies at the international and national levels that can be used to promote common child-friendly policies [40].

According to the results of our study, genetic factors are among the situational factors that can be the source of some developmental disorders and defects in children. The analysis of the effect of genes and the environment on the transmission of antisocial behaviors from parents to children, depression and hyperactivity shows that both genetics and family environment play a role in this regard [51]. The results of various studies have shown a vigorous relationship between early adverse conditions and epigenetic changes in genes related to stress responses, immunity, and the increase of mental disorders [56]. For example, based on the results of Roth’s study, early infant ill-treatment was associated with decreased expression of genes responsible for appropriate serotonin required to preserve mood balance [57]. In this regard, Vaida states that the integrated nature of growth and development is largely preserved through constant interactions between genes, hormones, nutrients, and other factors. Some of these factors that affect physical function are rooted in heredity. Factors such as season, dietary restrictions, and severe psychological stress are rooted in the environment. Other factors, such as the socioeconomic class, reflect a complex combination of hereditary and environmental effects which are likely to play a role throughout development [44]. Many studies have also emphasized the negative effects of air pollution as a situational factor on pregnant mothers and children. In this regard, Pem states in his study that fetuses that are exposed to lead and arsenic before birth may be born prematurely or at a low birth weight, and as a result, this can affect the development of the child [52].

## Conclusion

ECD focuses on equity and reducing the gap between rich and poor from the early years. Inequity in socioeconomic conditions will adversely affect the integrated development of early childhood, and children ‘s lack of optimal development will lead to the continuation of this unfavorable cycle. This principle is very weak in the current policies and programs of the country. Fair promotion of economic, cultural and social conditions of the society and consequently of the families can be very helpful in ECD and achieving the sustainable development of the society. While the context of our country is facing many challenges such as increasing social harms, reducing social capital, lack of public awareness, increasing socio-economic inequities, reducing economic growth, economic instability, etc. this will provide conditions for the abuse and neglect of children or their unfair growth and development. We should, therefore, consider creating integration between policies and programs of different sectors, prioritizing children in the welfare umbrella, empowering families, raising community awareness, and expanding services and support for families, specially the deprived families subject to special subsidies. Finally, we recommend that further studies be conducted on ECD in Iran including a survey of developmental disorders and delays in children and their relationship with social determinants of health, designing and surveying indicators in early care and education and support areas of children such as quality of early care and education, play, children with special needs, poverty, abuse, neglect, domestic violence, discrimination, children street, toxic stress and etc., conducting an evaluation/review of progress in reducing inequalities in various aspects of ECD, assessing the knowledge, attitude and practice of parents in relation to ECD in rural and urban areas, and examining the pilot implementation of ECD policy and its consequences in order to provide policy solutions.

### Strengths and limitations

This study was the first of its kind in conducting a deep and extensive analysis of social determinants of ECD in Iran. The results of the current study can improve the developmental conditions of children and lead to more attention to contextual factors in formulating policies related to ECD. However, our study has two main limitations; first, we have not presented the developmental status of children in various areas of ECD in the form of figures due to the lack of statistics and information in this field in our country. Second, some participants were not able to participate in the confirmation process because of their busy schedule and the lack of time.

### Policy recommendations

**Table:** 

**Dimension of solutions**	**Policy options**	**Proxy indicators for monitoring**
**Policy making considerations**	Paying attention to the context of the country in formulating policies	Is there strategic plan on ECD based on context of the country in the national level
	Increasing flexibility in national policies for children and families to address regional problems, needs and existing inequities	Is there strategic plan on ECD in the sub national level based on context of the regional problems
	Adhering to the standards of a child-friendly city in the design of urban spaces	Is there any policy on the standards of a child-friendly city in the design of urban spaces
	Amending and developing protective laws with executive guarantees in different areas including sexual abuse, trafficking, psychological abuse, neglect of children and etc.	Rate of child abuse (By different types of abuse)
**Promote Early Learning**	Establishing training courses of parenting and family and service providers’ empowerment	Rate of training courses of parenting and family annually
	Providing free and available education services with high quality to children in early years	The amount of free educational services provided to children
	Promoting advocacy from influential people in the field of children and child-friendly media and groups	Number of institutions and organizations about child support and child-friendly groups
**Integrated Interventions**	Creating coherence and integration between the policies of the various sectors involved in ECD	Number of ECD policies that have been formulated cross-sectorally
	Utilizing the capacity of NGOs and the private sector in policy making and implementation	Number of private institutions and NGOs in policy making and implementation of ECD
	Establishing an institution or body at a high level to coordinate and monitor the affairs of children such as the Children’s Commission in the Islamic Consultative Assembly or the Ministry of Children	Is there an institution at the highest level to coordinate and monitor children’s affairs?
	Visiting homes to screen, identify, evaluate, and track the children at high risk and improper conditions and take remedy actions depending on the identified problem, such as counseling with parents, parenting education, helping parents to quit addiction, financial aids, introduction them to the welfare organization and etc.	Number of screenings performed to identify, assess and track children at risk and adverse conditions
	Requiring health attachments for macro policies and programs in various fields with emphasis on the health of pregnant mothers and children	Are there health attachments to policies and programs on the health of pregnant mothers and children
**Promote financial supports in national level**	Developing a universal service package for children and families	Is there any social services package for children and families?
	Prioritizing children in the welfare umbrella and paying attention to children in support of low-income families	How many budget allocated to children in the welfare umbrella
	Allocating a certain share of the country’s GDP to children under 8 years of age.	Children under 8 years of age expenditure as GDP(%)
	Providing subsidies or tax exemptions to child care providers	The percentage of subsidies or tax exemptions to child care providers (%)

## Supplementary Information

**Additional file 1.**

## Data Availability

The data of this study are raw data which were accessible to the researchers in the interviews and are reported in the paper. The datasets used and/or analyzed during the current study are available from the corresponding author on reasonable request.
