# Supplementary material for: The impact of social determinants of health on early childhood development: a qualitative context analysis in Iran
Source: BMC Public Health. 2022 Jun 8;22:1149. doi: 10.1186/s12889-022-13571-5 (PMC9178833; doi:10.1186/s12889-022-13571-5)
Supplement: Supplementary file 1 — Additional file 1. [file 12889_2022_13571_MOESM1_ESM.docx]

**The impact of Social Determinants of Health on Early Childhood Development: A qualitative context analysis in Iran**

**Authors:** Omolbanin Atashbahar^1^, Ali Akbari Sari^2,3^, Amirhossein Takian^2, 4,5^, Alireza Olyaeemanesh ^5^, Efat Mohamadi^5^, Sayyed Hamed Barakati^6^

1. Department of Public Health, Sirjan School of Medical Sciences, Sirjan, Iran.
2. Department of Health Management and Economics, School of Public Health, Tehran University of Medical Sciences, Tehran, Iran.
3. National Institute for Health Research, Tehran University of Medical Sciences, Tehran, Iran.
4. Department of Global Health and Public Policy, School of Public Health, Tehran University of Medical Sciences, Tehran, Iran.
5. Health Equity Research Centre (HERC), Tehran University of Medical Sciences, Tehran, Iran.
6. Population, Family and School Health Office, Ministry of Health and Medical Education, Tehran, Iran.

Corresponding author**:** Efat Mohamadi, Health Equity Research Centre (HERC), Tehran University of Medical Sciences, Tehran, Iran. Email: Efat.Mohamadi @gmail.com, Mobile: 09120084809, Address: Health Equity Research Centre (HERC), No. 70, Bozorgmehr Ava., Vesal St., Keshavars Blvd., Tehran, Iran. Postal Code: 1416833481.Tel:02162921333.

**Appendix One: Interview Guide**

1. What factors in situational category (irregular and unstable events such as war) affect ECD and policymaking in various levels of micro, meso, exo and macro in Iran?
2. What factors in social category affect ECD and policymaking in various levels of micro, meso, exo and macro in Iran?
3. What factors in cultural category (values ​​of society or different groups in society) affect ECD and policymaking in various levels of micro, meso, exo and macro in Iran?
4. What factors in economical category affect ECD and policymaking in various levels of micro, meso, exo and macro in Iran?
5. What factors in political category affect ECD and policymaking in various levels of micro, meso, exo and macro in Iran?
6. What factors in international category (factors outside the national system of politics such as multinational corporations) affect ECD and policymaking in various levels of micro, meso, exo and macro in Iran?
7. What other factors do you think might affect early childhood development?
8. What works to decrease the existing inequities and improve the context for optimal early childhood development?
9. What changes or modifications (structural or other modifications) do you think is necessary for the ECD program to be successful?
10. If you had full authority in policymaking and making changes in this regard, what interventions did you give priority to in order to improve health, policymaking, and research?

**Appendix 2: Participants’ characteristics**

| **Interviewed Organization** | **Activity domains** | **Number of participants** | **Sex** | **Age range** |
| --- | --- | --- | --- | --- |
| Ministry of Health | Deputy of Social Affairs (SDH Administration, Social Harms Office, | 3 | Male | 50-60 |
|  |  |  | Male | 40-50 |
|  |  |  | Female | 30-40 |
|  | Population, Family, and School Health Office (Children’s Health Office and Infants’ Health Office) | 3 | Male | 50-60 |
|  |  |  | Female | 40-50 |
|  |  |  | Female | 30-40 |
|  | Ex manager of Nutrition Improvement Office | 1 | Female | 70-80 |
|  | Ex Minister | 1 | Male | 70-80 |
|  | Ex Health Deputy of Ministry of Health | 1 | Male | 70-80 |
| State Welfare Organization of Iran | Deputy of Social Affairs | 2 | Male | 50-60 |
|  |  |  | Female | 30-40 |
|  | Children and Adolescents’ Affairs Office | 1 | Female | 30-40 |
|  | Ex Director of Children and Adolescents’ Affairs Office | 1 | Male | 40-50 |
|  | Disability Prevention Office | 1 | Female | 50-60 |
| Ministry of Education | Preschool Office | 2 | Female | 40-50 |
|  |  |  | Male | 40-50 |
|  | Preschool Development and Planning Office | 2 | Female | 40-50 |
|  | Deputy of Physical Education and Health | 1 | Male | 50-60 |
| Ministry of Cooperatives, Labour, and Social Welfare | Direct supports and compensatory activities, Collaboration in ECD project | 1 | Female | 30-40 |
| Ministry of Interior | Deputy of Social Affairs, Collaboration in ECD project | 1 | Female | 40-50 |
| University of Social Welfare and Rehabilitation Sciences | Member of Iranian Society of Pediatrics, Pediatrician, ex manager of Disability Prevention Office of State Welfare Organization of Iran | 1 | Female | 50-60 |
|  | Member of SDH Research Center | 1 | Male | 30-40 |
|  | Neonatologist, Member of Neonatal Development Committee of Ministry of Health | 1 | Female | 50-60 |
|  | Pediatrician, Member of Pediatric Neurorehabilitation Research Center, Collaboration in ECD project | 1 | Female | 40-50 |
| Tehran University of Medical Sciences | Deputy of Social Affairs | 1 | Male | 50-60 |
|  | Pediatrician, Member of Iranian Society of Pediatrics | 1 | Male | 70-80 |
|  | Reproductive Health, Maternal and Child Health, Ex manager and executive officer in health centers of Ministry of Health | 2 | Female | 40-50 |
|  |  |  | Male | 50-60 |
|  | Health policymaking and management | 1 | Male | 50-60 |
| Judicial system of Iran | Judge | 1 | Male | 40-50 |
| Institute for the Intellectual Development of Children and Young Adults | Cultural and Artistic Creations Center | 1 | Male | 70-80 |
|  | Research Deputy | 1 | Male | 60-70 |
| DONYA Children’s Research Institute | Member of Board of Directors | 1 | Female | 70-80 |
| Society for Protecting the Rights of the Child (SPRC) | Member of Board of Directors | 1 | Female | 70-80 |
| The Parliament of Iran | Support from Children and Young Adults Fraction | 1 | Male | 60-70 |
| Ministry of Justice | National Body on the Convention on the Rights of the Child (CRC) | 1 | Male | 40-50 |
| Tehran Municipality | Health Office | 1 | Male | 40-50 |
| Children’s Medical Center | Health service provider, Member of Growth and Development Research Center | 1 | Male | 60-70 |
| High Council of Insurance | Secretariat of High Council of Health Insurance | 1 | Male | 40-50 |
